# Research Progress on *Macrococcus*: From Basic Biology to Clinical Antimicrobial Resistance Challenges

**DOI:** 10.3390/pathogens15060578

**Published:** 2026-05-27

**Authors:** Chenyu Zhan, Mingyu Zhang, Guijuan Hao, Yue Zhang, Fangkun Wang

**Affiliations:** 1 Shandong Agricultural University & Shandong Provincial Key Laboratory of Zoonoses, College of Veterinary Medicine, Shandong Agricultural University, Tai’an 271017, China; 18263620948@163.com (C.Z.); 19953187496@163.com (M.Z.); haogj2020@sdau.edu.cn (G.H.); 2 Department of Clinical Laboratory, The Affiliated Taian City Centeral Hospital of Qingdao University, Tai’an 271000, China

**Keywords:** *Macrococcus*, molecular characteristics, pathogenicity, antibiotic resistance, animal-derived pathogen, resistome

## Abstract

*Macrococcus* is a genus of Gram-positive cocci in the Staphylococcaceae family and a close phylogenetic relative of *Staphylococcus*. It is not a significant human pathogen but is known to widely colonize different environments, including animal skin and food products. Phylogenetically, *Macrococcus* is distinct from yet closely related to *Staphylococcus*, particularly the sciuri group. The species is effectively identified through such molecular markers as hsp60 and 16S rDNA. A key biochemical feature is an identified FAD-dependent oleate hydratase in *Macrococcus* equipercicus (*M. equipercicus*). Critically, *Macrococcus* carries various mobile antibiotic-resistance genes, especially against β-lactams (e.g., *mecB*, *mecD*) and macrolides (e.g., mef(F), msr(G)); these genes are located on plasmids, SCCmec-like elements, or resistance islands (e.g., McRI*_mecD_*), which facilitates their horizontal transfer. Surveillance confirms the widespread presence of methicillin-resistant *Macrococcus*, often with a multidrug-resistant phenotype, in food animals and their products. Although its own pathogenicity is low, *Macrococcus* acts as a reservoir and transmission platform for resistance genes: through horizontal gene transfer, it can potentially confer resistance to pathogenic *Staphylococcus*, thereby posing a threat to animal and public health. This review summarizes the basic biological characteristics and drug resistance-related research progress of the genus *Macrococcus*; it aims to provide a reference for subsequent studies as well as to establish technical support and a theoretical basis for the epidemiological investigation, drug-resistant strain identification, and clinical drug-resistance risk prevention and control of *Macrococcus*.

## 1. Introduction

### 1.1. Discovery of the Genus Macrococcus

The discovery and taxonomic classification of the genus *Macrococcus* involved a long iterative process from morphological observation to molecular biological verification, and the historical context of its separation from the genus *Staphylococcus* is closely related to the development of microbial classification technology. The genus’s taxonomic journey began in the late 19th century, when microbiologists conducted preliminary classifications of Gram-positive cocci through morphological observations; specifically, Ogston first named the cluster-forming cocci “*Staphylococcus*” in 1882 [1]. (Details on the literature retrieval and selection criteria regarding studies published since its discovery are described in Section 1.2.) While research on *Macrococcus* remains very limited, studies have found that *Macrococcus* are Gram-positive, coagulase-negative, catalase-positive, and oxidase-positive cocci that belong to the *Staphylococcaceae* family [2].

The purpose of this review is to provide a comprehensive synthesis of the current research progress on the genus *Macrococcus*, from its historical taxonomic classification to its emerging role in clinical settings. The scope of this article covers the fundamental biological and metabolic characteristics of its major species, their diverse ecological niches [3,4,5,6], and their evolving pathogenic potential in both veterinary and human medicine. Additionally, we critically evaluate the molecular mechanisms underlying its antimicrobial resistance and its significant function as a mobile reservoir for novel resistance genes, such as *mecB* and *mecD* [7]. By identifying current knowledge gaps and methodological limitations, this review aims to establish a theoretical basis for future epidemiological surveillance and for the development of effective control strategies against this emerging opportunistic pathogen.

### 1.2. Search Strategy and Selection Criteria

**Figure 1:**
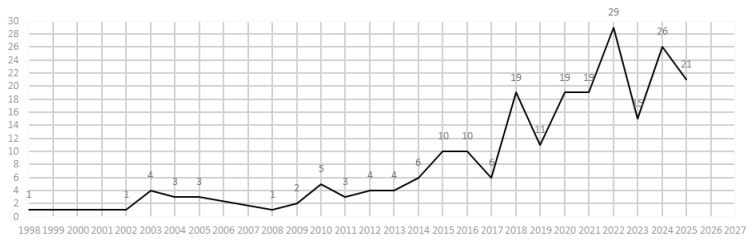
The annual number of published studies on *Macrococcus*.

Data for this review were identified by searches of PubMed, Web of Science, and Google Scholar. The search terms included “*Macrococcus*”, “molecular characteristics”, “pathogenicity”, and “antibiotic resistance”. Articles published from 1998 (the year of the formal proposal of the genus) to 2026 were considered (Figure 1). The selection included peer-reviewed original research and review articles published in English. Priority was given to studies involving clinical antimicrobial resistance, novel species identification, and horizontal gene transfer mechanisms. A total of 198 articles indexed on PubMed (as of the search date) formed the primary basis for the literature analyzed in this review.

## 2. Biological Characteristics

### 2.1. Morphological and Cultural Characteristics

**Figure 2:**
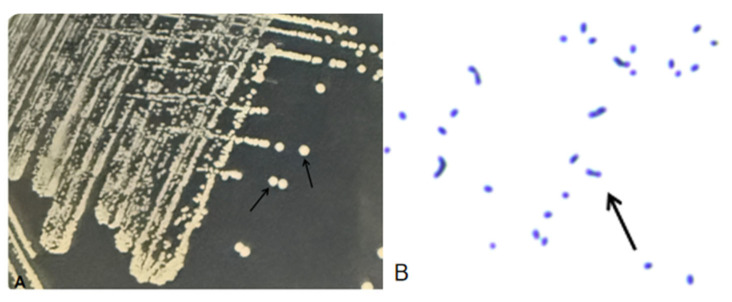
Characterization of the *Macrococcus*. (**A**): The isolated strain forms transparent yellow circular colonies on standard LB medium. (**B**): The isolated strain has a spherical morphology. The arrows indicate the colonies. Gram staining procedure: (1) Smear preparation: An autoclaved glass slide was placed on a clean bench, allowed to air-dry, and degreased over the outer flame of an alcohol lamp. A drop of ddH_2_O was placed on the slide using a flamed inoculation loop. A single colony was then picked and mixed with the ddH_2_O. Once air-dried, the smear was heat-fixed over the alcohol lamp flame. (2) Primary staining: An ammonium oxalate crystal violet solution was applied to the fixed smear for 1 min, followed by rinsing with distilled water to remove excess stain. (3) Mordanting: An iodine solution was applied for 1 min, then rinsed with distilled water. (4) Decolorization: The smear was decolorized using 95% ethanol (30 s in summer, 1 min in winter) and washed with water. (5) Counterstaining: A fuchsin solution was applied for 1 min. The slide was rinsed with distilled water, allowed to air-dry, and examined under a microscope.

*Macrococcus* species are Gram-positive cocci with a diameter of 1.1–2.5 μm, depending on the species and the growth medium. The cells are non-motile, non-sporulating, and typically arranged in pairs and tetrads, although single cells or short chains may occasionally be observed. When cultured on a standard LB medium, *Macrococcus* forms pale-yellow, circular, convex, translucent colonies with smooth surfaces and entire margins (Figure 2A). Gram staining reveals blue, spherical cells arranged in clusters, chains, or singles (Figure 2B). Members of this genus are facultative anaerobes [8] and generally test positive for catalase activity, with some strains also exhibiting oxidase-positive traits [9,10].

### 2.2. Genetic Classification of Macrococcus

Genome-level characteristics reveal the evolutionary patterns and potential functional differentiation of the genus *Macrococcus*. Currently, sequenced *Macrococcus* strains show typical genome sizes ranging from 2.0 to 2.5 Mb [11,12]: for instance, the *M. capreoli* genome is stably maintained between 2.4 and 2.5 Mb [7], while the complete genome of *M. bovicus* strain LI0213 (isolated from cattle) consists of a 2,082,488-bp chromosome and three plasmids [13]. Specifically, for *Macrococcus* strains from livestock, the gene pools carried by different populations vary significantly: one population contains genes of the β-lactamase family and genes related to toxin-antitoxin systems, while another population carries genes associated with the type VII secretion system [14]. Comparative genomics analyses have revealed significant population structure differences within the genus *Macrococcus*, with studies indicating that some species (e.g., *M. caseolyticus*; *M. armenti*) are composed of multiple intraspecific populations that exhibit distinct differentiation in their functional potential [14,15,16,17]. This phenomenon suggests that the pangenome of the genus *Macrococcus* has a certain degree of plasticity [14,18]. Notably, the accessory genome of *Macrococcus* is enriched with diverse mobile genetic elements, the detailed resistance mechanisms of which will be discussed in Section 4 [19,20,21,22,23].

This function of a “gene transfer station”, by transferring genes to more pathogenic *Staphylococcus* species (e.g., *Staphylococcus aureus*) via these diverse mobile genetic elements [9,24,25], significantly elevates the risk of antibiotic resistance dissemination. Consequently, monitoring *Macrococcus* in food, environmental, and clinical samples is crucial [2,26]. Furthermore, comprehensively elucidating the genus’s biological characteristics and genetic mechanisms holds important theoretical and practical significance related to addressing the potential future challenges of antibiotic resistance and to formulating effective scientific prevention and control strategies [27].

In terms of cultural characteristics and species identification, new species of the genus *Macrococcus* have low requirements for culture media and can grow on conventional bacterial media, including nutrient agar and sheep blood agar; however, significant differences between the species exist in colony morphology, hemolytic properties, and salt tolerance, providing important bases for species identification [15]. For example, *M. caseolyticus* is a Gram-positive, non-motile, non-spore-forming bacterium with a spherical or coccobacillary shape [11,12,16] that is commonly found in fermented foods (e.g., cheese, sausages) and animal-related environments (e.g., animal skin) [17,18], indicating that it can grow well under aerobic or microaerophilic conditions. With regard to its hemolytic properties, most *M. caseolyticus* strains do not exhibit hemolysis on sheep blood agar [12]; in terms of salt tolerance, some strains cannot grow at NaCl concentrations of 9% and 12% [11], and some newly discovered species show lower salt tolerance (<7.5% NaCl) [7] (Table 1).

### 2.3. Metabolic Classification of Macrococcus

Metabolic characteristics reflect the genus *Macrococcus*’s adaptability to different hosts and environments. Its metabolic activities, which exhibit distinct host adaptability and strain specificity, are focused primarily on carbon source utilization and energy metabolism.

From the perspective of energy acquisition, all strains of *Macrococcus* are heterotrophic microorganisms, which primarily obtain energy through the decomposition of organic carbon sources. In terms of temperature adaptability, the optimal growth temperature of some strains has been clarified: for instance, the optimal growth temperature of *M. capreoli* is 37 °C [7], in that the activity of enzymes related to energy metabolism reaches its peak at this temperature, which allows it to adapt to mesophilic environments. Although the optimal growth temperature of most strains has not been determined, their isolation sources are mostly the body surfaces or interiors of animals (e.g., horses, dogs, humans) [8,9,12]; thus, it can be inferred that all strains are adapted to mesophilic environments, with temperatures ranging from 30 to 37 °C.

The difference in carbon source utilization is another important manifestation of the metabolic characteristics of *Macrococcus* strains, as well as one of the core bases for strain identification. *Macrococcus* strains isolated from animals and *M. armenti* can utilize D-ribose, and the latter can also ferment sucrose [8,11]; most strains can also utilize common carbohydrates such as glucose. At the same time, *Macrococcus* strains isolated from horses cannot utilize methyl-β-D-glucopyranoside or maltose [8], and *M. hajekii*, *M. bohemicus*, and *M. lamae* are all unable to produce acid from glycerol [10]. Due to their low salt tolerance (<7.5% NaCl), the carbon source utilization of *Macrococcus* strains isolated from roe deer may be inhibited in high-salt environments, but no special restrictions in their carbon source utilization profiles have been found in conventional low-salt environments [7].

The diversity in carbon source utilization profiles among *Macrococcus* species is summarized in Table 2, which highlights key biochemical reactions that are useful for species-level identification.

### 2.4. Taxonomic Status of Macrococcus

The genus *Macrococcus* is closely related to the genus *Staphylococcus*. It is traditionally not considered a human pathogen but rather tends to be classified as an associated veterinary pathogen, and it is widely present in animal-derived foods [2].

The genus’s expanding species inventory raises a fundamental question: how was the genus *Macrococcus* established and distinguished from its close relative *Staphylococcus*? The answer lies in the progressive refinement of molecular taxonomic tools.

Historically, the 16S rRNA gene has served as a foundational molecular marker for bacterial classification [21]. Differences in 16S rRNA gene sequences are sufficient to differentiate the genus *Macrococcus* from *Staphylococcus*—a distinction that has proven vital in clinical settings. For instance, in cases involving immunocompromised patients where routine proteomic tools (like MALDI-TOF MS) fail due to database limitations, 16S rRNA sequencing has successfully identified emerging species like *M. canis*, which reinforces the independent taxonomic identity of the genus [21].

Furthermore, the development of next-generation sequencing has enabled core genome analysis, which provides a more robust basis for bacterial classification. Methods like calculating average nucleotide identity (ANI) and digital DNA-DNA hybridization (dDDH) using multiple housekeeping genes have provided definitive genetic evidence for the distinction of *Macrococcus* from *Staphylococcus* at the whole-genome level, thereby allowing for precise species-level identification [8].

Notably, the taxonomy of the *Staphylococcaceae* family has undergone significant revisions in recent years, which has important implications for understanding the evolutionary position of *Macrococcus*. In 2020, a comprehensive phylogenomic analysis led to the reclassification of the *Staphylococcus sciuri* group (including *S. sciuri*, *S. fleurettii*, and related species) into the novel genus *Mammaliicoccus* [22]. On the phylogenetic tree, *Mammaliicoccus* occupies an intermediate position between *Macrococcus* and the core *Staphylococcus* clade, indicating that *Macrococcus* represents a more deeply branching lineage within the family. Clarifying this taxonomic relationship holds practical importance, as *Mammaliicoccus* has been frequently identified as an intermediate host and evolutionary conduit for the transmission of resistance genes—most notably *mecB*—from *Macrococcus* to *S. aureus* [23].

### 2.5. Ecological Niches and Host Associations of Macrococcus

Members of the genus *Macrococcus* exhibit a remarkably broad ecological distribution, given their isolation from a wide range of animal species and environmental reservoirs. As identified in Table 2, *Macrococcus* strains are commonly recovered from the skin and mucosal surfaces (e.g., nasal cavities) of various domestic animals, including cattle, pigs, horses, dogs, and cats [3,6,8,11]. In addition to livestock and companion animals, *Macrococcus* species have been identified in wild animals, such as roe deer and red deer [7], as well as in processed food products, including cheese and traditional fermented meats [4,5,17,18]. This extensive range of hosts and habitats underscores the ecological versatility of the genus.

While *Macrococcus* primarily persists as a commensal colonizer of animal surfaces, there is a growing recognition that certain species can transition to an opportunistic pathogenic lifestyle under conducive host conditions. The clinical significance and the virulence potential of *Macrococcus* in both veterinary and human medicine are examined in detail in the following section.

### 2.6. Clinical Diagnosis and Identification of Macrococcus

The accurate clinical diagnosis of *Macrococcus* infections remains a significant challenge, which frequently leads to underreporting.

Phenotypic Identification: Traditional automated biochemical systems (e.g., VITEK) routinely misidentify *Macrococcus* species as coagulase-negative staphylococci (CoNS), due to overlapping phenotypic and metabolic profiles [12,28].

Proteomic Analysis: MALDI-TOF MS is currently the gold standard for identification; however, its diagnostic accuracy is heavily contingent on the comprehensiveness of the reference database. If clinical laboratories rely on databases lacking recent spectral profiles for emerging species like *M. canis* or *M. goetzii*, isolates will remain unidentified or misclassified [12,21,29,30].

Molecular and Genomic Diagnostics: Molecular techniques are essential to overcoming phenotypic limitations. 16S rRNA gene sequencing is highly effective for differentiating *Macrococcus* from *Staphylococcus*. Additionally, species-specific PCR targets, such as the *ctaC* gene, have been developed to rapidly discriminate *M. caseolyticus* and *M. canis* from closely related staphylococci. Whole-genome sequencing (WGS) is increasingly utilized for definitive species confirmation and resistome profiling [8,14].

### 2.7. Genotyping and Molecular Epidemiology

Understanding the population structure and the transmission dynamics of *Macrococcus* relies heavily on advanced genotyping methods. Comparative genomics and core genome analyses (utilizing ANI and dDDH) have become standard for resolving the complex phylogenetic relationships within the *Staphylococcaceae* family [8]. Genotyping reveals significant genomic plasticity within the *Macrococcus* pangenome, with strain-specific distribution of mobilomes, prophages, and genomic islands [14]. This high level of horizontal gene transfer means that WGS is increasingly necessary not just for species identification but also for tracking the epidemiological spread of specific multidrug-resistant clones across the animal–human–environment interface [8].

## 3. Pathogenicity and Clinical Significance of *Macrococcus*

### 3.1. Pathogenic Role of Macrococcus in Animals

*Macrococcus* typically exists as commensal bacteria in animals, but in recent years it has been increasingly identified as an opportunistic pathogen, and it has played a particularly significant role in veterinary clinical infections [19]. Among the *Macrococcus* species involved in veterinary clinical infections, *M. caseolyticus* and canine-derived *Macrococcus* are common species that are often associated with opportunistic infections, such as bovine mastitis [19,31,32]. For example, studies have isolated methicillin-resistant *M. caseolyticus* strains from the milk of cows with mastitis; these strains carry novel methicillin-resistance genes (e.g., *mecD*) located on a mobilizable chromosomal island (McRI*_mecD_*), indicating their potential threat to udder health [11,31].

In terms23 of pathogenic mechanisms, *Macrococcus* can express a variety of virulence factors, including genes encoding hemolysins, adhesion proteins, biofilm formation, exotoxins, and capsule synthesis. These factors enhance the bacteria’s adhesion and invasion capabilities, which in turn promote the establishment and persistence of infections [33]. For instance, a highly pathogenic *M. caseolyticus* strain (SDLY) isolated from commercial broiler chickens was shown to form a distinct capsule around itself, and experiments confirmed that it can cause significant clinical symptoms and histopathological changes, such as inflammatory infiltration and multifocal necrosis [24]. Biofilm formation is particularly important, as it helps bacteria resist host immunity and antibiotic treatment, thereby playing a key role in chronic infections such as mastitis [32].

In addition to dairy cows and broiler chickens, *Macrococcus* also causes infections in other animals. In dogs, multiple case reports have isolated *M. canis* and *M. caseolyticus* from otitis, rhinitis, and skin-infection sites [3,34,35]. For example, a screening study of 162 dogs found that 13 dogs carried *M. canis* and 6 carried *M. caseolyticus*; multiple strains were obtained from infected sites, and these strains often carried methicillin-resistance genes such as *mecB*, which underscores their importance in companion animal health [3,35]. In addition, *M. bohemicus* isolated from canine otitis also carried a novel *mecB* gene, further confirming the diversity and clinical relevance of *Macrococcus* in canine infections [34]. Although few cases in the current literature explicitly involve horses, the broad host adaptability of *Macrococcus* as an animal-derived pathogen suggests that it may also pose potential infection risks for other livestock.

In summary, through its virulence factors and resistance mechanisms, *Macrococcus* acts as an opportunistic pathogen in animals that causes various infections (bovine mastitis being a typical example) and is frequently detected in dogs. Therefore, enhanced monitoring and prevention in veterinary practice are necessary [3,19,31].

### 3.2. Potential Role in Human Infections

While *Macrococcus* is traditionally regarded primarily as a harmless commensal of animal skin and food products rather than a primary human pathogen, an expanding body of literature increasingly challenges this view. Recent evidence indicates that *Macrococcus* is an emerging opportunistic pathogen capable of bridging the gap between animal reservoirs and human clinical settings. The clinical reality presents a stark contrast to the historical assumption, necessitating a paradigm shift.

*Macrococcus* is not only an opportunistic pathogen causing veterinary infections like bovine mastitis and canine otitis [3,19], but it also demonstrates the capacity to cross host barriers and threaten human health. For instance, novel species such as *M. goetzii* have been isolated directly from human clinical specimens [30], and *M. caseolyticus* has even been co-isolated in severe, life-threatening human polymicrobial necrotizing fasciitis [25]. Pathogenic threat genomic analyses revealed putative virulence factors in novel species and subspecies, including fibronectin-binding proteins and intracellular proteases [30]. Furthermore, clinical case reports have documented *Macrococcus* strains successfully inducing human infections, such as severe human skin and wound infections (e.g., associated with methicillin-resistant *M. canis* and *M. bovicus*) [13]. This emerging clinical relevance is further substantiated by a number of documented cases.

The transmission routes of *Macrococcus* involve contact with bacteria-carrying animals or consumption of contaminated animal-derived foods. On this point, studies have shown that methicillin-resistant *Macrococcus* has been detected in retail meats in Switzerland (e.g., beef and pork), indicating that the food chain may serve as an important route for human exposure [11]. Furthermore, multi-drug-resistance genes (e.g., *mecB*, *mecD*, and macrolide-resistance genes) carried by animal-derived strains may be transferred to human pathogens (e.g., *S. aureus*) via mobile genetic elements, which further increases the public health risks [3].

Multiple clinical case reports have documented the isolation of *Macrococcus* from human infection samples. Jost first isolated a methicillin-resistant *M. canis* strain LI021 from a human skin-infection wound; this strain carried the *mecB* gene located on a novel pseudo-SCC*mec* element, confirming that *M. canis* not only exists in animals but can also survive in human tissues and may be involved in the infection process [20]. In a case of human necrotizing fasciitis, *M. caseolyticus* was co-isolated with other bacteria, indicating its potential role in polymicrobial infections [25]. Additionally, reports have shown that the plasmid-borne *mecB* gene has been transmitted to *S. aureus* strains isolated from human clinical samples, leading to resistance to β-lactam drugs; this further confirms the dual role of *Macrococcus* as both a “reservoir” of resistance genes and a potential pathogen in human infections [27].

While historically viewed as an animal commensal, clinical evidence increasingly implicates *Macrococcus* in specific human pathologies. The most frequently documented clinical manifestations involve skin and soft tissue infections (SSTIs). For instance, methicillin-resistant *M. canis* has been directly isolated from human skin infection wounds, demonstrating its ability to colonize and infect human tissue. Furthermore, *Macrococcus* has been implicated in severe, life-threatening conditions; notably, *M. caseolyticus* was co-isolated in a case of human polymicrobial necrotizing fasciitis. This suggests that while *Macrococcus* may lack the aggressive virulence to routinely cause severe monomicrobial infections in healthy individuals, it can act as a significant co-pathogen in complex, polymicrobial environments, complicating the clinical picture and treatment trajectory.

### 3.3. Virulence Factors of Macrococcus

Through genomic studies, *Macrococcus* (especially *M. caseolyticus* and canine-derived *Macrococcus*) has been identified as carrying a variety of potential virulence-associated genes, which indicates that it possesses a certain degree of pathogenic potential [33]. The virulence factors encoded by these genes include adhesins (e.g., fibronectin-binding proteins), proteases, hemolysins (e.g., *hlgB* and *hlgC*), immune evasion proteins (e.g., capsular polysaccharide synthases), and exotoxins [24,36].

In the highly pathogenic *M. caseolyticus* strain SDLY, an eight-gene capsular polysaccharide synthesis gene cluster was identified; the transferases and synthases it encodes promote capsule formation and enhance the bacteria’s resistance to host immune clearance [24]. Similarly, in *M. canis*, putative γ-hemolysin genes (*hlgB*, *hlgC*) and the methicillin-resistance gene *mecB* coexist on mobile genetic elements, indicating that virulence and drug resistance may undergo coordinated evolution and transmission [20,26].

The ability to form biofilm is another key virulence trait of *Macrococcus*, and it is closely associated with the establishment of persistent infections on host tissue surfaces or medical devices. Studies have shown that the genome of methicillin-resistant *M. caseolyticus* contains genes encoding biofilm formation-related proteins [33]. Biofilms not only help bacteria adhere to biological or abiotic surfaces but also significantly reduce the bacteria’s susceptibility to antibiotics by forming a physical barrier, thereby leading to treatment failure and chronic infections. This trait is particularly important in opportunistic pathogens, as highlighted by Cotting et al. [3]: “The presence of canine-derived *Macrococcus* at infection sites and its antibiotic resistance emphasize that more attention should be paid to this emerging bacterial species.” Specifically, biofilm-mediated persistence and tolerance are non-negligible pathogenic mechanisms. Therefore, an in-depth understanding of *Macrococcus* virulence factors (especially its biofilm-forming ability) is crucial for evaluating their clinical significance and for formulating effective infection control strategies.

In addition to specific virulence factors, the regulatory mechanisms controlling virulence expression are also key determinants of pathogenic potential. Unlike *S. aureus*, *Macrococcus* appears to lack a functionally intact and homologous accessory gene regulator (*agr*) quorum-sensing system. In *S. aureus*, the *agr* system coordinates the transition from a colonization state to an invasive state, marked by massive toxin secretion. The absence or divergence of this key regulatory network in *Macrococcus* may explain why, despite carrying multiple virulence genes, it primarily behaves as a commensal organism in immunocompetent hosts and only exhibits opportunistic pathogenicity under specific conditions [27].

In its interaction with the host immune system, *Macrococcus* exhibits relatively weak immune-evasion capacity. Although certain highly pathogenic strains (e.g., *M. caseolyticus* SDLY) can resist phagocytosis via capsule, most *Macrococcus* lack key surface proteins like *S. aureus* protein A (SpA). SpA specifically binds to the Fc region of immunoglobulins, thereby blocking antibody-mediated opsonophagocytosis. The absence of this core immune evasion mechanism renders *Macrococcus* more susceptible to clearance by the host’s innate immune system, limiting its ability to cause severe systemic infections [24,33].

### 3.4. Risk Factors for Human Colonization and Infection

Based on the ecological niche and transmission routes of *Macrococcus*, several key risk factors for human colonization and infection can be identified:

Occupational Exposure: Individuals working closely with livestock (farmers, veterinarians, and abattoir workers) have an elevated risk of exposure to animal-adapted strains (e.g., *M. bovicus*, *M. armenti*) [11].

Companion Animal Contact: Pet owners, particularly those handling dogs with chronic otitis or skin conditions, face exposure risks to *M. canis* and *M. caseolyticus* [3,34,35].

Foodborne Exposure: The frequent detection of methicillin-resistant *Macrococcus* in retail meats and cheeses indicates that the handling and consumption of contaminated undercooked animal-derived foods is a potential pathway for colonization [11,20,28].

Host Immune Status: As an opportunistic pathogen with relatively weak intrinsic immune-evasion capabilities, human infections are more likely to occur in individuals with compromised skin barriers (e.g., surgical wounds, trauma) or preexisting immunosuppression [21,24,33].

## 4. Antimicrobial Resistance of *Macrococcus*: Current Status, Mechanisms, and Transmission Risks

### 4.1. Overview of Resistant Phenotypes

Although *Macrococcus* is not a traditional human pathogen, its widespread presence in animals, food, and the environment—coupled with its various antimicrobial-resistance genes—has drawn attention from the public health sector. Multiple studies have shown that *Macrococcus* isolates from clinical and environmental sources exhibit varying degrees of resistance to a range of antimicrobials, with resistance to β-lactam antibiotics being the most prominent.

Among the *Macrococcus* strains from food-producing animals and meat products, the methicillin-resistant phenotype is relatively common. In a survey conducted in Switzerland, Keller et al. found that methicillin-resistant *Macrococcus* (predominantly *M. caseolyticus*) isolated from calf nasal swabs exhibited co-resistance not only to β-lactam drugs but also to tetracycline (carrying the *tet(L)*, *tet(K)*, and *tet(M)* genes), streptomycin (carrying the *str* and *ant(6)*-*Ia* genes or *rpsL* mutations), kanamycin (carrying the *ac(6′)-Ie-aph(2″)-Ia* gene), clindamycin (carrying the *erm(B)* and *erm(45)* genes), erythromycin (carrying the *erm(B)*, *msr(G)*, and *erm(45)* genes), fusidic acid (carrying the *fusC* gene), and gentamicin (carrying the *aac(6′)-Ie-aph(2″)-Ia* gene) [11].

In canine-derived *Macrococcus*, a study by Cotting et al. showed that both canine-derived *Macrococcus* and *M. caseolyticus* were detected on canine skin and at infection sites. Among the antimicrobial-resistance profiles of these isolates, methicillin resistance mediated by the *mecB* gene was the most common, which reinforces the prevalence of this resistant phenotype in animal hosts [3]. Furthermore, a study on the pathogenic *M. caseolyticus* strain SDLY isolated from commercial broiler chickens demonstrated that it exhibited multidrug resistance (MDR), thereby confirming that bacteria of this genus can accumulate and express a broad range of resistance determinants under specific conditions [24].

Notably, methicillin-resistant *Macrococcus* is also frequently isolated from the food chain in, e.g., retail cheeses and raw meat products. These strains not only show resistance to β-lactams but also often exhibit concurrent resistance to multiple antimicrobials, including macrolides, tetracyclines, and aminoglycosides—in other words, displaying characteristics of MDR [20,28]. Collectively, these epidemiological data reveal the potential role of *Macrococcus* as a resistance gene reservoir, and the diversity of its resistant phenotypes poses complex challenges for clinical treatment and food safety control.

### 4.2. In-Depth Analysis of Major Resistance Mechanisms

The antimicrobial resistance of *Macrococcus* is mediated by a variety of complex molecular mechanisms that are typically carried by mobile genetic elements, which not only provide the bacteria themselves with strong adaptability but also pose a significant risk of transmitting resistance to pathogenic staphylococci (e.g., *S. aureus*). The resistance mechanisms of *Macrococcus* vary depending on the type of drug (Table 3).

Moreover, as a large and mobile reservoir of antibiotic resistance genes, *Macrococcus* carries novel methicillin resistance genes such as *mecB* and *mecD*. These genes are mobilized via various mobile genetic elements, most notably staphylococcal cassette chromosome mec (SCC*mec*) elements and plasmids. Unlike the classical staphylococcal *mecA* gene—which is typically restricted to chromosomal SCC*mec* elements—*mecB* is highly mobile and is often housed within mobilome-like elements, large conjugative plasmids (which share 99.96% identity with *S. aureus* plasmids), and specific cassette elements [26]. For example, the novel transposon-defective SCC*mec*_KM45013_ element isolated from *M. canis* is 39-kb in length, integrates at the 3′ end of the chromosomal orfX gene, and contains 49 coding sequences. Its structural features include imperfect direct repeats acting as integration site sequences, core cassette chromosome recombinase genes (*ccrAm2* and *ccrBm2*), and a *mec* gene complex (*mecRm-mecIm-mecB-blaZm*) [12]. Additionally, human clinical isolates have revealed novel SCC*mec*-like elements containing a unique mec gene complex, representing a ‘missing link’ in SCC evolution [9]. Both *mecB* and *mecA* encode low-affinity penicillin-binding proteins that mediate resistance to β-lactam antibiotics; however, novel *Macrococcus mec* genes exhibit distinct resistance profiles. Specifically, *mecD* confers resistance to all classes of β-lactams, including the anti-MRSA cephalosporins ceftaroline and ceftobiprole, to which staphylococcal strains carrying the *mecA* gene often remain susceptible [37]. This highlights a more comprehensive resistance profile than those conferred by *mecA*, *mecB*, and *mecC*. Epidemiologically, the prevalence of *mecB* is highly significant: it is the most common resistance phenotype observed in both colonizing and clinically infecting canine-derived *Macrococcus* isolates [3], and is widespread across the food chain, frequently colonizing food-producing animals and contaminating retail meats [11].

In short, *Macrococcus* possesses a large and evolving reservoir of resistance genes, which are often located on mobile genetic elements such as plasmids, transposons, and integrative islands. Furthermore, its close evolutionary relationship with staphylococci facilitates the transfer of these resistance genes—particularly novel *mec* genes—to more pathogenic staphylococcal species (including *S. aureus*), thereby posing a persistent threat to human and animal health.

### 4.3. Transmission of Resistance Genes and Public Health Risks

The primary public health concern regarding *Macrococcus* lies in its role as a reservoir for antimicrobial-resistance genes, particularly due to the potential for their horizontal transfer to pathogenic bacteria like *S. aureus* [39].

#### 4.3.1. Potential for Horizontal Gene Transfer

Abundant molecular evidence indicates active gene exchange between *Macrococcus* and other Gram-positive bacteria, such as *S. aureus*. This transfer is primarily mediated by the mobile genetic elements of plasmids, transposons, and integrative chromosomal islands.

Among the various mobile genetic elements mediating this exchange plasmid-mediated gene transfer provides the clearest evidence for direct genetic flow between these two genera, because plasmids—as independent and complete replicative unit—can autonomously transfer between different bacterial cells and achieve stable inheritance. Studies have found that the multidrug-resistant plasmid pKM0218, which carries the *mecB* gene and was isolated from canine-derived *Macrococcus*, is nearly identical (99.96% nucleotide identity) to the *mecB*-harboring plasmid identified in clinical *S. aureus* isolates; this strongly indicates that recent plasmid exchange has occurred between the two genera [26]. However, it is important to note that while plasmid-mediated transmission is a prominent feature here, the *mecB* gene is not exclusively plasmid-borne: as discussed earlier, it is broadly distributed across both chromosomal cassettes (e.g., SCC*mec*) and extrachromosomal genetic elements (e.g., plasmids) [12,26]. Similarly, large *mecB*-carrying plasmids have been identified in a newly discovered psychrophilic *Macrococcus* species, and in vitro conjugation experiments have confirmed that these plasmids can be conjugatively transferred to *S. aureus* strains [9].

Cross-genera integration potential of chromosomal islands: *Macrococcus*-specific resistance islands, such as McRI*_mecD_* (which carries *mecD*), have site-specific integrases that have been shown to precisely integrate model DNA elements into the *rpsI* gene locus on the chromosomes of *S. aureus*, *Staphylococcus pseudintermedius*, and even *Bacillus* species under experimental conditions [37]. This indicates that such islands possess the molecular potential to spread across intergeneric barriers under favorable conditions. The novel antimicrobial resistance gene *mecD* encodes an alternative penicillin-binding protein 2a (PBP2a) that confers a broader spectrum of resistance to β-lactam antibiotics—including the latest generation of anti-MRSA cephalosporins—compared to staphylococcal *mecA* and *mecC*, as well as *mecB* [37]. Unlike *mecA* and *mecC*, which are typically located on staphylococcal cassette chromosome *mec* (SCC*mec*) elements mobilized by serine recombinases, *mecD* is uniquely situated on *Macrococcus*-specific genomic resistance islands, such as McRI*_mecD_*-1 and McRI*_mecD_*-2, and is entirely independent of SCC*mec* or the Tn6045 transposon [36]. These chromosomal islands feature a 5′ tyrosine recombinase integrase gene (int) and divergently oriented intR and xis open reading frames, exhibiting a phage-like genetic organization similar to *Staphylococcus* aureus pathogenicity islands (SaPIs) [36]. Crucially, this specific genetic architecture endows these chromosomal islands with significant cross-genera integration potential. Under experimental conditions, the site-specific integrases of McRI*_mecD_* have been shown to precisely integrate model DNA elements into the 3′ end of the 30S ribosomal protein S9 gene (*rpsI*) locus on the chromosomes of *Staphylococcus aureus*, *Staphylococcus pseudintermedius*, and even *Bacillus* species [37]. This indicates that such islands possess the powerful molecular machinery required to spread across intergeneric barriers, thereby facilitating the horizontal transfer of broad-spectrum β-lactam resistance to major pathogens. Furthermore, because *mecD* shares only about 63% homology with *mecA*, it can lead to false negatives in conventional PCR testing, complicating clinical detection and public health surveillance [37].

Sharing of other resistance genes: Beyond *mec* genes, macrolide resistance genes (e.g., the *mef(D)*–*msr(F)* operon) have been found in highly similar genetic contexts (e.g., the SaRImsr island) in both *Macrococcus* and *S. aureus*. This further corroborates the continual and extensive gene flow between these two genera [33].

#### 4.3.2. Role as a Resistance Gene Reservoir or Amplifier

A core insight emerges based on the above-outlined mechanisms. *Macrococcus* likely acquires and accumulates a variety of resistance genes from the environment or from commensal microbiota in animal hosts (e.g., dogs, cattle, pigs) and along the food production chain. Since these resistant strains typically do not cause severe disease, they can persist and proliferate under antibiotic selection pressure. When they coexist with pathogenic staphylococci in the same ecological niche (e.g., animal nasal cavities, skin infection sites, contaminated meat), their “arsenal” of resistance genes—especially *mecB* and *mecD*, which confer broad-spectrum resistance to clinically critical β-lactams—can be transferred to more pathogenic bacteria (e.g., *S. aureus*) via mobile elements. Once this gene transfer occurs, it can convert methicillin-sensitive *S. aureus* (MSSA) into methicillin-resistant *S. aureus* (MRSA) as well as endow existing MRSA strains with additional or enhanced resistance. This significantly exacerbates the challenges to clinical antibiotic treatment.

On this point, reports have documented the detection of *mecB* originating from *Macrococcus* in clinical isolates of *S. aureus* from both humans and animals, marking the successful “spillover” of genes from this reservoir to major pathogens [25]. Consequently, monitoring *Macrococcus* in animals and food is no longer merely a veterinary or food safety issue but a critical component of public health, in line with the One Health framework. In other words, controlling the prevalence and transmission of resistance genes in *Macrococcus* is crucial for slowing the rate of failure of clinically important antibiotics, particularly β-lactams.

### 4.4. Current Methodological Limitations and Knowledge Gaps

Despite advances in characterizing the genomics and resistome of *Macrococcus*, significant methodological limitations and knowledge gaps remain in the field. Diagnostically, leading to widespread clinical underreporting [12,28]. Even MALDI-TOF MS, the current gold standard, is strictly limited by the comprehensiveness of its reference databases; the absence of recent spectral profiles for emerging species like *M. canis* inevitably leads to misclassification [12,21,30]. Furthermore, conventional PCR testing may yield false negatives for the novel *mecD* gene because it shares only approximately 63% homology with the classic *mecA* gene, complicating public health surveillance [37].

Critical gaps also persist regarding the genus’s pathogenicity and transmission mechanisms. While genomic studies identify multiple virulence genes, *Macrococcus* lacks a functionally intact agr quorum-sensing system [27], which leaves a major gap in our understanding of how it coordinates virulence expression to transition into an invasive state. Crucially, although in vitro conjugation experiments have confirmed that large plasmids and chromosomal islands carrying genes like *mecB* can transfer to *S. aureus* [26,36], the absolute frequency of in vivo horizontal gene transfer in animal or human microbiomes under varying antibiotic selective pressures remains completely unknown.

## 5. Conclusions and Future Directions

This review stresses a critical ecological and clinical paradigm shift regarding the genus *Macrococcus*. No longer merely a benign environmental commensal or food fermentation agent, *Macrococcus* is emerging as a clinically relevant opportunistic pathogen and, more importantly, a highly adaptable reservoir for novel antimicrobial-resistance genes (such as *mecB* and *mecD*). This scientific synthesis of the current literature reveals that the genomic plasticity of *Macrococcus*—driven by diverse mobilomes, including large conjugative plasmids and phage-related chromosomal islands (e.g., McRI*_mecD_*)—equips it with a potent molecular arsenal. While the absolute frequency of in vivo horizontal gene transfer to high-consequence pathogens like *S. aureus* remains to be fully quantified, the structural homologies and in vitro transferabilities strongly suggest that *Macrococcus* serves as a crucial evolutionary stepping stone for resistance genes. Its widespread presence across the One Health spectrum (food animals, retail meats, companion animals, and occasionally humans) underscores its role as a persistent mobile threat to the efficacy of critical β-lactam antibiotics.

To effectively mitigate the risks posed by *Macrococcus*, future research and clinical interventions must directly address the current methodological limitations and knowledge gaps in several actionable directions. First, diagnostic laboratories must urgently update their MALDI-TOF MS reference databases to include emerging species (e.g., *M. canis*, *M. armenti*), to resolve phenotypic misidentification issues and reveal the true clinical burden. Furthermore, epidemiological studies must move beyond isolated sampling to implement longitudinal, WGS-based surveillance. This is essential for tracking the co-colonization and genomic exchange dynamics between *Macrococcus* and *Staphylococcus* species in shared ecological niches, such as dairy farms and abattoirs. Additionally, it is urgently needed for future mechanistic studies to utilize animal microbiome models to accurately quantify the in vivo horizontal gene transfer frequencies of *mecB* and *mecD* under varying antibiotic selective pressures. Finally, given the genus’s widespread prevalence in veterinary settings, research should actively explore targeted decolonization strategies (such as *Macrococcus*-specific bacteriophage therapy or competitive exclusion probiotics) to proactively deplete this resistance gene reservoir in livestock before zoonotic spillover occurs.

## Figures and Tables

**Table 1:** Core Biochemical Reaction Characteristics of Different Strains.

Strain Name	Core Biochemical Reaction Characteristics	Key Identification Points	References
*M. equipercicus*	D-ribose metabolism negative Lacks α-glucosidase activity Unable to ferment methyl-β-D-glucopyranoside to produce acid Unable to ferment maltose to produce acid	No clear characteristic	Belhout C et al., 2025 [8]
*M. animalis*	D-ribose metabolism positive Able to ferment methyl-β-D-glucopyranoside	Ability to ferment methyl-β-D-glucopyranoside is a key distinguishing characteristic from *Macrococcus equi*.	Belhout C et al., 2025 [8]
*M. capreoli*	Catalase test positive Oxidase test positive Intrinsic resistance to fosfomycin	No clear distinguishing characteristic	Schiffer CJ et al., 2025 [7]
*M. armenti*	Able to ferment D-ribose Does not produce DNAse No hemolysis Able to ferment sucrose Possesses α-glucosidase activity	Ability to ferment sucrose is a distinguishing characteristic from *Macrococcus caseolyticus* subsp. caseolyticus. Possession of α-glucosidase activity is a distinguishing characteristic from *Macrococcus caseolyticus* subsp. hominis.	Keller JE et al., 2022 [11]
*M. canis*	Most strains exhibit visible hemolysis on sheep blood agar The DNA-DNA relatedness with the type strain of *Macrococcus* caseolyticus is only 53.7%	No clear distinguishing characteristic	Gobeli Brawand S et al., 2017 [12]
*M. caseolyticus* subsp. hominis	α-Glucosidase activity absent	Absent α-Glucosidase activity is a key distinguishing characteristic from *Macrococcus vitulinus*.	Mašlaňová I et al., 2018 [9]
*M. hajekii*, *M. bohemicus*, *M. lamae*	Resistant to bacitracin Susceptible to furazolidone Able to produce phosphatase Most strains are able to reduce nitrate Unable to ferment glycerol to produce acid	No clear distinguishing characteristic (share core biochemical reaction characteristics)	Mannerová S et al., 2003 [10]

**Table 2:** Currently recognized *Macrococcus* species and their reported isolation sources.

Macrococcus species	Isolated from	References
*M. caseolyticus*	Cattle	Kloos et al., 1998 [4]
*M. equipercicus*	Horses	
*M. bovicus*	Cows, Horses	
*M. carouselicus*	Horses	
*M. brunensis*	Llamas	Mannerová S et al., 2003 [10]
*M. hajekii*		
*M. lamae*		
*M. canis*	Dogs	Gobeli et al., 2017 [12]
*M. bohemicus*	Humans	Maslanova et al., 2018 [19]
*M. epidermidis*		
*M. goetzii*		
*M. armenti*	Pigs, Calves	Keller et al., 2022 [11]
*M. capreoli*	Deer	Schiffer et al., 2025 [7]
*M. psychrotolerans*	Animals, Humans	Mašlaňová et al., 2025 [9]
*M. animalis*, *M. equine*	Horse, Pigs, Cattle, Cats	Belhout et al., 2025 [8]

**Table 3:** Summary Table of Drug-Resistance Mechanisms of *Macrococcus* to Different Classes of Antibiotics.

Resistance Category	Main Resistance Mechanism	Key Gene/Protein	Core Characteristics/Research Basis
β-lactams	Produce low-affinity penicillin-binding proteins (PBP2A homologs) Produce β-lactamases	*mec* gene family: *mecB*, *mecD* *blaZ* series: *blaZm*	*mecB*: First identified in *Macrococcus caseolyticus* and located on the chromosome SCC*mec*: Mobilome-like elements or large plasmids, capable of conjugative transfer in vitro to *Staphylococcus aureus* [36] *mecD*: Located on the *Macrococcus*-specific resistance island McRI*_mecD_*, it exhibits a broader resistance spectrum than *MecB* and staphylococcal *MecA*, has potential for intergeneric transmission [37], shares approximately 63% homology with *mecA*, and may lead to false negatives in conventional PCR *blaZm*: Encodes penicillinase and often forms the mecI-mecR1-mec-blaZ complex with the *mec* gene
Macrolide-Lincosamide- Streptogramin B (MLSB) class	Ribosomal target site modification Active efflux	*erm* gene: *erm (B)*, *erm (C)*, *erm (T)*, *erm (43)*, *erm (44)*, *erm (45)*, *erm (48)* efflux protein gene: *mef (F)-msr (G)* operon, *mef (D)-msr (F)* operon	*erm* gene: Encodes a ribosomal methyltransferase that methylates specific adenine residues of 23S rRNA, mediating MLSB-type cross-resistance [3] *mef(F)-msr(G)*: Plasmid-borne, encodes an efflux pump and an ABC-F type ribosomal protection protein, mediates the MS phenotype (macrolide-resistant but lincomycin- and streptogramin B-susceptible) [38] *mef(D)-msr(F)*: Located on a resistance island, present in *Macrococcus* and *Staphylococcus aureus*
Tetracyclines	Ribosomal protection Active efflux	*tet(M)* (ribosomal protection protein) *tet(L)* (membrane-associated efflux pump)	Genes are mostly located on mobile genetic elements, facilitating interbacterial transmission [11]; *tet(L)* can actively efflux tetracyclines
Aminoglycosides	Aminoglycoside-modifying enzymes modify the drug to inactivate it	Acetyltransferase: *aac (6′)-Ie-aph (2″)-Ia* Phosphotransferase: *aph (2′)-Ib, aph (2′)-Ic, aph (3′)-IIIa3.* *nucleotidyltransferase*: *ant (4′)-Ia, ant (6)-Ia*	Through chemical modification of aminoglycosides by different types of modifying enzymes, the drugs are inactivated
Fluoroquinolones	Chromosome-related gene mutations leading to low drug affinity	*gyrA, grlA* (staphylococcal homologous genes)	Mutations in the gene reduce the affinity of DNA gyrase and topoisomerase IV for fluoroquinolones; this mechanism has been identified by sequencing the quinolone resistance-determining region (QRDR) of canine *Macrococcus* isolates [3]

## Data Availability

No new data were created or analyzed in this study.
